# Eco‐Friendly and Ready‐To‐Market Polyurethanes: A Design of Experiment‐Guided Substitution of Toxic Catalyst and Fossil‐Based Isocyanate

**DOI:** 10.1002/cssc.202402451

**Published:** 2025-01-17

**Authors:** Gabriele Viada, Nicole Mariotti, Simone Galliano, Alberto Menozzi, Claudia Barolo, Matteo Bonomo

**Affiliations:** ^1^ Department of Chemistry NIS Interdepartmental Centre and INSTM Reference Centre University of Turin Via G. Quarello 15 A 10135 Torino Italy; ^2^ Demak Polymers Corso Lombardia 44 10151 Torino Italy; ^3^ Institute of Science, Technology and Sustainability for Ceramics National Research Council of Italy Via Granarolo 64 48018 Faenza Italy; ^4^ ICxT Interdepartmental Center University of Turin Lungo Dora Siena 100 10153 Torino Italy.

**Keywords:** Polyurethanes, Design of Experiment, Circular Economy, Green catalysts, Bio-based polymers

## Abstract

In this contribution, we tackle the replacement of the Hg‐based catalyst and fossil‐derived isocyanate precursors toward the formulation of a more sustainable polyurethane thermosetting resins (PUs), emulating the performance of a fully fossil‐based one employed in industrial encapsulation of optoelectronics. A mixed Bi−Zn catalyst and a 71 % bio‐based isocyanate are exploited at this aim through multivariate chemometric approaches, namely Design of Experiment (DoE). DoE allows us to investigate the effect of different formulation factors on selected parameters, such as the film flexibility and transparency or the gel time. More in detail, it is found that a low amount of Zn‐rich catalytic mixture leads to a ready‐to‐market polyurethane only when a fossil‐based isocyanate is used. Differently, PUs formulated with bio‐based isocyanate, albeit showing a higher bio‐based content, present an insufficient optical purity, jeopardizing their market acceptability. Nevertheless, adding a negligible amount of a specific long chain fatty acid as reactivity modulator in the formulation leads to a bubbles‐free and ready‐to‐market resin showing an impressive 65 % w/w content of circular and bio‐based components.

## Graphical abstract

Increasing bio‐based and circular components in polyurethane formulation up to 65 %w/w maintaining the market acceptability through multivariate analysis approach

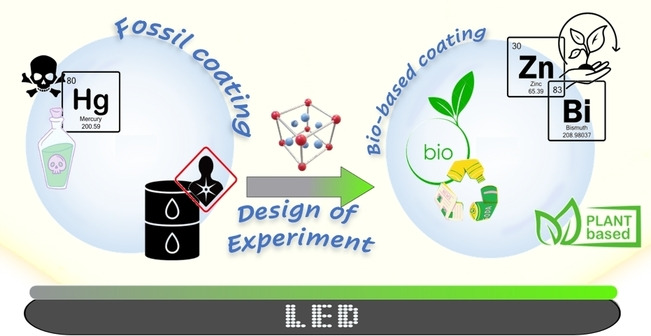

## Introduction

In recent years, the use of polyurethanes (PUs) has increased enormously due to their versatile synthesis and tunable physicochemical properties ranging from soft flexible coatings to hard rigid materials. Nowadays, polyurethanes are used in different realities, from building construction and automotive to optical devices and photovoltaics as encapsulant and edge‐sealant materials.[^1^, ^2^, ^4^, ^81^] The synthetic pathway leads to linear thermoplastic polyurethanes (TPUs) or cross‐linked thermosetting polyurethanes, depending on the precursors′ functionality (number of reactive units).^[5]^ The ease in tailoring the chemical and mechanical properties of PUs has produced an increasing interest not only in the scientific community but also in the industrial context: in 2021, global PUs production reached almost 25 Mt and a Compound Annual Growth Rate (CAGR) close to 6 %.^[6]^

Even though the rise of polyurethane materials, the conventional synthetic pathway of PUs (*i. e*., a polyaddition between di‐ or polyisocyanates and di‐ or polyols in the presence of a catalyst) presents some dramatic sustainability concerns: precursors used in the synthesis of both polyols and isocyanates come mostly from petroleum‐based sources, thus leading to adverse environmental impacts and poor sustainability. Moreover, when dealing with thermosetting PUs, an additional issue concerns their End‐of‐Life: the only industrially available alternative to landfilling is the burning of the polymer, leading to additional CO_2_ production and further negatively impacting the material sustainability.[^7^, ^8^] In this context, the European Union has been recently committed to the “High Ambition Coalition to End Plastic Pollution” project,^[9]^ pushing both industries and academic research to innovate the whole PUs life‐cycle, from the raw materials selection to the end‐of‐life disposal.[^10^, ^11^, ^12^] Here, bio‐based and waste‐derived precursors can be valid candidates for more sustainable and circular PUs that comply with market requirements.[^13^, ^14^] In this perspective, in our previous work, the fossil‐based polyol of a commercial thermosetting polyurethane for C.A.S.E.applications has been partially replaced with BHET (bis(2‐hydroxyethyl) terephthalate) and Sovermol780, as waste‐derived and bio‐based components, respectively, maintaining the market‐desired characteristics in terms of transparency, thermal and long‐term stability.^[3]^ Despite the remarkable achievements (*i. e*. 55.5 %w of sustainable input in the polyols mixture and 24.4 %w in the final PU formulation), the mercury‐based catalyst and 100 % fossil‐based isocyanate employed still represent two critical issues in the winding path toward a truly sustainable polyurethane formulation.

As regards catalysts, organotin and organomercury compounds are well known for accelerating the urethane reaction and still represent one of the most common catalytic systems for polyurethane reactions in many industrial realities. Mercury‐based catalysts (*e. g*. phenylmercury neodecanoate) show remarkably high selectivity toward the isocyanate‐polyol reaction and allow excellent processability of the resin (*i. e*. a long enough induction period coupled with a short curing time).[^15^, ^16^] Thus, they have been widely used in the formulation of elastomer PU, sealants and coating.[^17^, ^18^, ^19^] On the other hand, tin‐based catalysts also showed promising properties, such as high catalytical activity and selectivity towards the urethane reaction, with dibutyltin dilaurate being one of the most widely used due to its excellent compatibility with both different solvents and the components of PU formulations. It should be noted that, from an efficiency and reactivity point of view, there are no reasons to consider a replacement of these catalysts. However, toxicity‐related issues are responsible for their phasing out.^[5]^ In this respect, the European Commission has progressively banned mercury‐based compounds since 2005^[20]^ whereas, on a worldwide scale, its adverse effects on human health^[21]^ and the environment[^22^, ^23^] are controlled by the Minamata Convention.[^24^, ^25^] Afterwards, the use of mercury‐containing catalysts in polyurethane production has been prohibited since 1^st^ January 2018.^[24]^ Similarly, also organostannates have been restricted over time: since 2009, tin‐based organic compounds classification changed in “toxic and environmental hazardous substance”^[26]^ and they have been classified as persistent organic pollutants. Their use is worldwide regulated by the International Maritime Organization, whereas in Europe by R.E.A.C.H.^[27]^ Considering the above‐mentioned issues, replacing toxic and critical raw material‐based catalysts with more sustainable ones cannot be postponed. In this context, many efforts have been made to find effective alternatives and alkali‐like catalyst, generally considered more eco‐friendly than organometallic compounds in terms of toxicity, environmental impact and regulatory restrictions, could be considered a valid alternative.[^28^, ^29^] However, while tertiary amines are useful for foam production, where rapid curing and bubble formation may be desirable, organometallic catalysts are still preferred in high‐performance polyurethane systems (e. g. C.A.S.E.) due to their superior selectivity, reaction control and efficiency.[^12^, ^30^, ^31^]

As a result, different metal‐based catalysts have already been investigated.[^32^, ^33^, ^34^] Magnesium and aluminium complexes were developed for their catalytic behaviour in polyurethane synthesis,^[35]^ while zirconium and titanium compounds were reported as efficient catalysts for preparing polyurethane foam.[^36^, ^37^, ^38^] However, these types of catalysts generally present moderate activity requiring to relatively high dosages, which contrast with the ninth principle of Green Chemistry[^39^, ^40^] and, thus, impacting the sustainability of the process.

Taking the cue from literature overview, bismuth and zinc‐based compounds appear to be the most viable candidates for PU formulations due to their low toxicity, high selectivity, and synergistic effect on the polymerization reaction. Indeed, they proved to be nearly equivalent to conventional Hg‐based catalysts.^[37,41][37,42–44]^ Bismuth derivatives are treated as non‐problematic from a toxicological point of view, but their catalytic activity appears challenging to be controlled.^[45]^ Moreover, organo‐bismuth systems are sensitive to moisture, decreasing the selectivity toward the isocyanate–polyol reaction and promoting side reactions, posing some challenges in the production of a market acceptable resin.[^16^, ^44^] On the other hand, zinc carboxylate compounds demonstrate lower activity than other catalysts, posing severe limitations on their employment as single catalysts.

Nowadays, different bismuth‐based and zinc‐based catalysts have been proposed, and are already commercially available and utilized all over the PUs‐industrial realities. However, due to the abovementioned challenges, the utilization of bismuth‐zinc mixtures could be seen as the only feasible approach to overcome the critical aspects of the catalysts when used individually.

Concerning isocyanates, whose harmful toxicological summary is reported in the ECHA registration dossier(“ECHA ‐ European CHemical Agency,” 2023), they are traditionally obtained from phosgene, an aggressive and lethal gas, and the two most widely used di‐isocyanates in PU industry, MDI (methylene diphenyl di‐isocyanate) and TDI (toluene di‐isocyanate), are 100 % fossil‐based and are classified as CMR (carcinogenic, mutagenic and reprotoxic).[^47^, ^48^] Furthermore, in February 2020, the REACH Committee also voted in favour of the European Commission′s proposal for a di‐isocyanate restriction.^[49]^

In recent years, several efforts have been made to find viable alternatives to isocyanates in PU formulations. Among these, NIPUs (non‐isocyanate polyurethane) have proved to be virtuous examples toward the complete replacement of isocyanates within polyurethane formulations.[^10^, ^50^, ^51^] However, most of them suffer from a significant lack of thermal stability and their lower reactivity prevents any industrial application..^[52]^ In this context, implementing bio‐based isocyanates from renewable sources as precursors represents a promising trade‐off between sustainability and market acceptability. Indeed, whereas NIPUs require many synthesis steps and show reduced reactivity,^[10]^ bio‐isocyanates are easier to produce and implement as precursors within the formulation process, thus being potentially attractive not only to academic but also to industrial realities.

Considering the aforementioned issues, this research work aims to replace toxic mercury‐based catalyst and fossil‐based isocyanate within a thermosetting and transparent polyurethane formulation employed in the optoelectronic field, maintaining its performances in terms of physical and optical properties toward an eco‐friendly and ready‐to‐market product. Firstly, the Hg‐based catalyst has been replaced with organobismuth and organozinc catalysts in a PU formulation based on a 100 % fossil‐derived isocyanate. The bismuth/zinc ratio and the total amount of catalysts into the formulation were studied to observe their influence on the final optical and thermal properties of polyurethane. Secondly, the fossil‐derived isocyanate has been replaced by a bio‐based alternative, obtained chiefly from renewable sources, in order to enhance the eco‐friendly components of polyurethane and be compliant with the European regulation.^[53]^ Considering the successful approach presented in our previous work,^[3]^ the Design of Experiment (DoE) has been exploited as a multivariate chemometric method to comprehensively evaluate the effects of such substitutions on the final optical and thermal properties of proposed polyurethanes. In particular, two similar DoEs have been performed on fossil‐based (DoE 1) and bio‐based (DoE 2) isocyanate, and the different outcomes have been deeply discussed and compared with a commercial reference PU formulation. Finally, the proposed formulations have been optimized to produce ready‐to‐market polyurethanes with comparable features to the commercial reference, while significantly enhancing the overall sustainability and eco‐friendliness of the PU resin.

## Results and Discussion

### Substitution of Hg‐Based Catalyst

Organobismuth (Coscat® 83) and organozinc (Coscat® Z‐22R) catalysts have been selected to substitute the mercury‐based catalyst. Coscat® 83 is a highly reactive catalyst that does not allow a long enough processability time since the polymerization causes too rapid increase in viscosity of the polyurethane blend. Contrary, Coscat® Z‐22R shows a significantly slower kinetics of the polymerization process, therefore it could be employed in combination with Coscat® 83 to avoid bismuth oxidation and its consequent inactivation promoted by air..[^37^, ^44^] Therefore, a mixture of these two catalysts is desirable to achieve high selectivity towards the urethane reaction, preventing the occurrence of side reactions.

Initially, explorative experiments have been conducted to determine suitable working ranges for the Bi‐ and Zn‐based catalysts, focusing on their relative ratio (Bi/Zn) and total concentration (C%) in the PU blend formulated with the fossil‐based isocyanate. C% between 0.1 % and 0.8 % showed good processability regardless of the B/Z ratio, resulting in easy‐to‐handle PU formulations. Contrary, C% lower than 0.1 % led to extremely long curing time, thus negatively impacting the industrial applicability of the final formulations. While C% higher than 0.8 % led to highly viscous PU mixtures characterized by an extremely fast curing kinetic, compromising the optical quality of the final film, i. e. high amount of bubbles inside the films. Indeed, the PU formulation process involves mechanical stirring of polyols and isocyanate, which can result in the incorporation of a considerable amount of air into the mixture. This is particular evident when rapid curing occurs, such as in the cases of high concentration of catalyst. However, the mechanical stirring might not be the sole source of bubbles inside the films. An important side reaction that could occur during PU polymerization regards the interaction between isocyanate and water. If moisture is present within the mixture, the isocyanate may react with it to generate an unstable carbamic acid, subsequently decomposing into urea and carbon dioxide.[^54^, ^55^] These byproducts can be entrapped into the polymeric film, thus resulting in foaming.[^56^, ^57^] This side reaction is particularly facilitated by highly exothermic reaction environment, which is promoted by an excessive increase in the amount of catalyst.

Hence, considering these results, a multivariate statistical approach based on DoE was preferred over a one‐factor‐at‐a‐time (OFAT) method to simultaneously evaluate the influence of C% and B/Z factors on various properties of the final PUs. A Full Factorial design with two variables at three levels have been selected for the multivariate investigation The lists of experiments conducted for DoE 1 is reported in Table 1, along with the corresponding measured responses.

**Table 1:** Experimental plan for DoE 1 adopted for PUs formulated with fossil‐based isocyanate.

	Factors			Responses			
Experiment	B/Z	C%	Run Order	T% [%]	Gel time [min]	T_g_ [°C]	Bubbles [a.u.]
**F1**	0.1	0.1	11	90.3	265	51.6	1
**F2**	0.5	0.1	5	89.4	135	63.0	1
**F3**	0.1	0.8	8	86.1	15	57.4	2
**F4**	0.5	0.8	10	23.2	3	62.7	4
**F5**	0.1	0.45	4	88.9	59	61.2	1
**F6**	0.5	0.45	9	86.0	7	64.0	2
**F7**	0.3	0.1	1	89.6	180	56.2	1
**F8**	0.3	0.8	7	77.4	7	58.4	3
**F9**	0.3	0.45	3	88.8	38	57.2	2
**F10**	0.3	0.45	6	88.2	40	63.3	2
**F11**	0.3	0.45	2	86.5	37	63.1	2

The replicated data (F9, F10, and F11) exhibit good reproducibility of measured responses, confirming the adequacy of the preparation process for PU films. The models obtained through the DoE analysis of the experimental results have been properly refined by removing not significant terms (at 90 % confidence level), and the resulting equations are reported as follows:
(1)

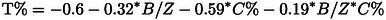



(2)

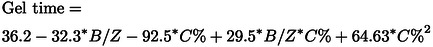



(3)

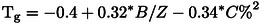



(4)

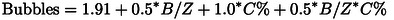




Of note, data distribution has been transformed using negative logarithms for T% (−log_10_(100‐1.1y)) and T_g_ (−log_10_(65‐y)) to reduce skewness and improve statistics. The computed models show satisfactory goodness‐of‐fit and predictive ability for T% (R^2^ 0.94, Q^2^ 0.81), gel time (R^2^ 0.99, Q^2^ 0.96), and bubbles (R^2^ 0.95, Q^2^ 0.89), in contrast to T_g_ model that exhibits lower values (R^2^ 0.71, Q^2^ 0.42) The analysis of the observed versus predicted values (Figure S1) indicates the lower data fit can be attributed to the relatively high variability among replicates (see experiment F9 vs F10 and F11) compared to the total variability of the entire experimental domain. Despite this evidence, the experiments of every response show good matching between observed and predicted values indicating good regression models. The ANOVA analysis further confirmed all models are statistically significant at 95 % confidence level (Table S1). Moreover, the analysis of the residuals versus the run order of experiments (Figure S2) does not highlight any dependence of the responses on time. Finally, every model has been successfully validated by comparing experimental and predicted values obtained from an additional randomized trial not comprised in the DoE plan (Table S2). The results indicate a satisfactory ability in describing and predicting response values within the investigated domain.

**Figure 1:**
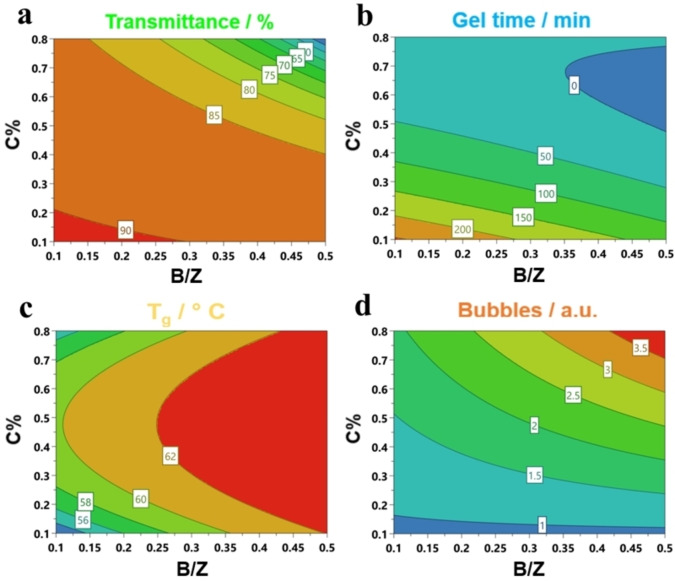
Isoresponse contour plots of DoE 1 for a) T%, b) Gel time, c) T_g_, and d) Bubbles.

To improve understanding of the reciprocal interaction between factors and responses, isoresponse counter plots have been generated according to the computed models (Figure 1). Considering the film transmittance (Figure 1a), polyurethanes formulated with lower amount of catalyst and B/Z ratio yield significantly higher T% values, even exceeding 90 %, which a commonly required for highly transparent coatings in the optoelectronic field. These formulative conditions also lead to longer induction time (gel time up to 200 min, Figure 1b), allowing a more effective degassing of resin with minimized entrapped air (amount of bubbles, Figure 1d). Consequently, this leads to high optical quality of the polyurethane film. On the contrary, formulations with high C% and B/Z values tend to accelerate the curing kinetics, resulting in dramatically shorter gel times (close to 0 min) and higher heat generation. This behaviour not only increases the incorporation of air inside PUs film but also promotes side‐reactions and formation of CO_2_ (bubbles close to 4 a.u.). The resulting polyurethanes are characterized by difficult workability and poor optical quality (Figure S3a), jeopardizing their industrialization and market acceptability.

Considering the glass transition temperature (Figure 1c), the use of low C% and B/Z ratio leads to the minimum values (~52 °C). Conversely, increasing the B/Z values, especially, enhance the rigidity of PU film (>62 °C), as also observed during preliminary experiments. This can be attributed to the exothermic nature of the polyurethane reaction:^[58]^ a higher amount of bismuth‐based catalyst leads to a greater heat release during polymerization, promoting the crosslinking and more extensive network formation in the PUs system.[^58^, ^59^] In conclusion, the DoE analysis highlights that manipulating the concentration and ratio of Bi/Zn catalysts allows for the proper tuning of the optical and physical properties of polyurethane film. The combination of bismuth and zinc catalysts appears to be effectively suitable for replacing toxic Hg‐catalyst in commercial polyurethanes based on fossil‐derived isocyanate.

### Substitution of Fossil‐Based Isocyanate

After demonstrating the possibility of replacing the mercury‐based catalyst in standard PU formulations, the research work has been focused on the substitution of the fossil‐based isocyanate with a bio‐based derivative, i. e. Desmodur N7300.Keeping the same factors, responses, and design selected for DoE 1, a second multivariate investigation has been performed to study the effect of different amounts and ratios of Bi/Zn catalysts when applied in PU formulation with bio‐based isocyanate. The lists of experiments executed for DoE 2 is reported in Table 2, along with the corresponding measured responses. Even in such a system, a good reproducibility of measured responses has been observed among replicated data (B9, B10, and B11). This evidence confirms the mixing compatibility between the catalysts and the bio‐based isocyanate used in formulation and the suitable preparation process adopted for PU films. The DoE models obtained through the analysis of the experimental results have been computed, and the corresponding equations are reported as follows:
(5)

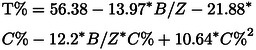



(6)

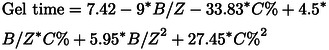



(7)

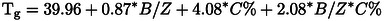



(8)

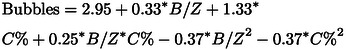




**Table 2:** Experimental plan for DoE 2 adopted for PUs formulated with bio‐based isocyanate.

	Factors			Responses			
Experiment	B/Z	C%	Run Order	T% [%]	Gel time [min]	T_g_ [°C]	Bubbles [a.u.]
B1	0.1	0.1	4	88.0	87	36.6	1
B2	0.5	0.1	2	89.0	61	35.0	1
B3	0.1	0.8	3	64.7	10	39.8	3
B4	0.5	0.8	8	16.9	2	46.5	4
B5	0.1	0.45	11	74.1	25	39.6	2
B6	0.5	0.45	6	37.1	5	39.7	3
B7	0.3	0.1	5	89.7	70	35.7	1
B8	0.3	0.8	1	53.8	3	45.5	4
B9	0.3	0.45	9	56.0	6	39.6	3
B10	0.3	0.45	7	59.1	6	39.8	3
B11	0.3	0.45	10	55.6	7	41.8	3

The response models exhibit excellent goodness‐of‐fit and predictive ability (T%: R^2^ 0.96, Q^2^ 0.76; Gel time: R^2^ 0.99, Q^2^ 0.98; T_g_: R^2^ 0.93, Q^2^ 0.75; Bubbles: R^2^ 0.98, Q^2^ 0.84). The ANOVA test (Table S5), the analyses of the Observed vs. Predicted values (Figure S4) and the Residuals vs. Run Order plots (Figure S5) further confirmed every model is good and significant at 95 % confidence level. Finally, the models have been validated by comparing experimental and predicted values of an additional, randomly selected, experimental trial (Table S2).

**Figure 2:**
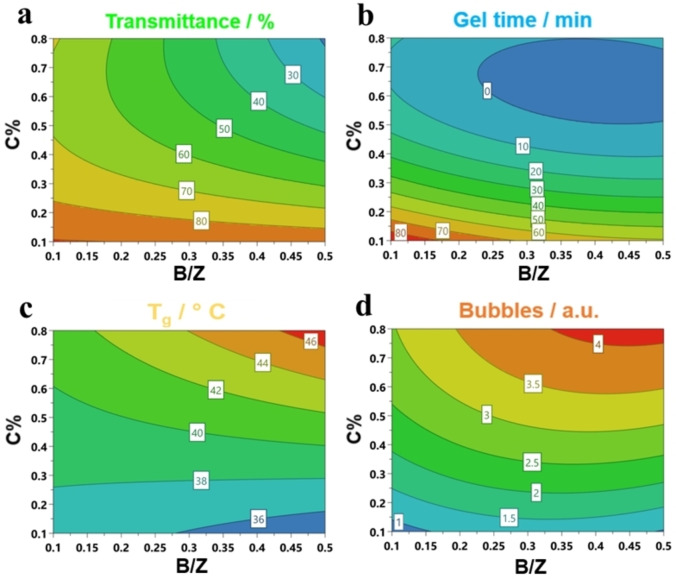
Isoresponse contour plots of DoE 2 for a) T%, b) Gel time, c) T_g_, and d) Bubbles.

Isoresponse counter plots calculated by models of DoE 2 (Figure 2) generally show similar interactions among factors and responses observed in DoE 1.

The main differences observed between DoEs are related to the response values themself. Considering the entire experimental domain, polyurethanes formulated with bio‐based isocyanate tend to have lower transmittance (Figure 2a) and a greater amount of bubbles (Figure 2d), particularly when high C% and B/Z ratio are used. Contrary, the gel time (Figure 2b) and glass transition temperature (Figure 2c) result markedly reduced by around 100 minutes (at low C% and B/Z) and 20 °C, respectively.

A possible explanation for these different outcomes could be related to the different NCO functionalities (2.2 for fossil‐based and 3.7 for bio‐based) and viscosity of the isocyanates. It is well‐established that a greater functionality increases both the viscosity and the curing speed of the formulations, leading to a shorter gel time..[^60^, ^61^] Indeed, the gel time is the time requested to reach a critical viscosity that determines the physical transition from *sol* to *gel*.^[62]^ Thus, the higher the starting viscosity of the formulation (modulated by the isocyanate), the shorter the time to reach the critical viscosity and, consequently, the shorter the gel time. Polyurethane resins from DoE 1 showed a viscosity (measured immediately after mixing polyols with isocyanate) of around 0.6 Pa*s, remarkably lower than the formulations from DoE 2 (10.5 Pa*s), and this is due to the much lower viscosity of fossil‐based isocyanate (0.45 Pa*s vs 9 Pa*s at r.t.). Additionally, the faster polymerization kinetics observed with the bio‐based isocyanate could also be explained by looking at the different molecular structures of isocyanate precursors. The fossil‐based DK180HV isocyanate is based on IPDI, an asymmetric di‐isocyanate, where the two NCO moieties are positioned differently: one is directly bound to the cyclohexane core, while the second one is linked through an additional methylene unit (Figure S6a).^[63]^ On the contrary, Desmodur Eco N7300 is a symmetric isocyanurate, where both functional groups are bound to the cycle through a five‐atom carbon chain (Figure S6b).^[52]^ In this case, once the first NCO moiety reacts, the polymerization of the others is not hindered, being linked at the end of a free‐to‐move aliphatic chain. In contrast, in DK180HV, the absent or shorter length of the chain results in hindered accessibility of the NCO groups. Furthermore, the higher viscosity and curing kinetic of bio‐based isocyanate system result in PUs film with greater amount of incorporated and reaction‐induced bubbles compared to fossil‐based polyurethanes. The relatively faster polymerization kinetic of bio‐based isocyanates has been also observed in the literature, for example, when Desmodur Eco N7300 is employed in place of L‐lysine ethyl ester di‐isocyanate or Tolonate X FLO 100.^[52]^

Regarding the flexibility (to be intended as the mechanical behavior observed on a macroscopic scale) of PU films, a clear correlation between the nature of the isocyanates and the T_g_ response has been observed. T_g_ values ranges from 51.6 to 64.0 °C and from 35.0 to 46.5 °C for the fossil‐based and bio‐based isocyanate, respectively. This behaviour could be explained considering (i) the different %NCO (i. e. percentage of free‐to‐react NCO groups on isocyanate) of Desmodur Eco N7300 (21.5 %) and DK180HV (27.0 %), and (ii) the selection of the hydroxyl:isocyanate ratio as a constant factor (*i. e*. 1) in the DoEs. These two conditions correspond to a different polyol:isocyanate weight ratio (Table 5). Indeed, with lower %NCO, the polyols:isocyanate ratio decreases, resulting in fewer urethane nodes per volume. This contributes to the higher flexibility observed in PUs based on bio‐based isocyanate.

The analyses of the above‐discussed data proved the promising properties of our bio‐based thermosetting polyurethanes as processable, transparent and flexible coating films.

### Optimization of Polyurethane Formulations

Aiming to develop a market‐ready thermosetting polyurethanes for encapsulating optoelectronics, a formulation with features comparable to commercial PU (Table 3) should be achieved. Therefore, we decided to exploit the predictive ability of DoE to identify specific PU formulations (one for each investigation) that would emulate or even ameliorate the features of the commercial resin.

**Table 3:** Properties of commercial and more eco‐friendly PU formulations.

Sample	C% [%]	B/Z [w/w]	T% [%]	Gel time [min]	T_g_ [°C]	Bubbles [a.u.]	Viscosity [Pa*s]	Bio‐based and recycled component^[b]^ [%]
Commercial PU^[a]^	0.215	‐	88.0	70	35.0	0	0.35	0
^[a]^PU_opt_	0.312	‐	88.0	38	55.0	0	0.58	24.4
DoE 1 PU_opt_	0.21	0.5	89.6	80	59.0	1	0.57	24.4
DoE 2 PU_opt_	0.11	0.2	89.3	78	35.3	1	10.5	65.0
DoE 2 PU_opt_ LCA	0.11	0.2	89.0	80	35.7	0	10.1	64.9

^[a]^ Formulated with Hg‐based catalyst; ^[b]^Calculated by weight.

The Sweet Spot plot is a very useful tool in looking for optimal formulations. It shows the area where the combination of factors leads to the achievement of one or more desired properties (Figure S7). Hence, in order to emulate the main physical‐chemical properties and reactivity of the reference commercial polyurethane, three requirements were set i) high transmittance with T%≥88 %, ii) perfect optical purity with no bubbles, and iii) suitable gel time in the range of 60–80 minutes. T_g_ response was not considered, as all the values obtained in the experimental domain from the DoEs are acceptable for industrial application in optoelectronics.

However, no PU formulations from DoE 1 and DoE 2 have been identified to achieve all three set requirements, affecting their industrial applicability. Specifically, the optical impurity (amount of bubbles=1) has emerged as the most challenging aspect, and the analyses highlighted the impossibility of achieving the ′zero bubbles′ condition through any combination of factors within the experimental domain for both fossil‐ and bio‐based formulations.

**Figure 3:**
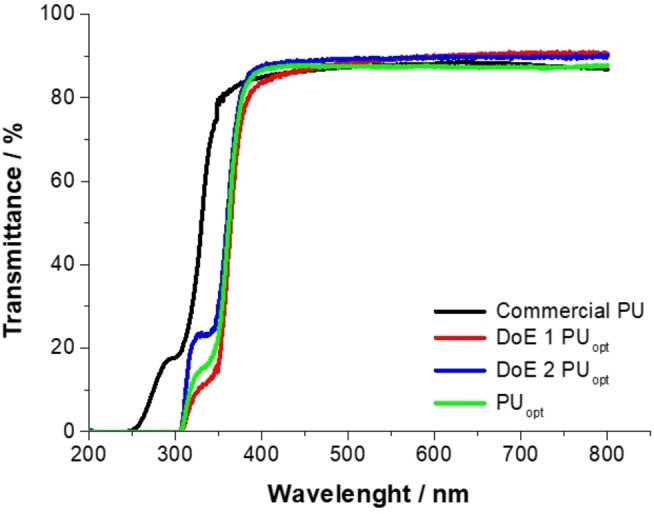
Transmittance of commercial and optimized PU films.

Given these premises, the software‐aided optimization of the PU formulations was performed to approach as closely as possible the industrial requirements. According to the desired specifications of the responses (*i. e*. T%≥88 %, the lowest amount of bubbles as possible and gel time from 60 to 80 min), the software helps to find the best combinations of factors. Among them, the formulations requiring the lowest C% have been selected (Table S3 and S4) in order to minimize the total amount of catalysts inside the PUs formulations and be compliant with the ninth principle of Green Chemistry.[^39^, ^40^] Following this evaluation, optimized resins has been formulated with C%=0.21 % or 0.11 % and B/Z=0.5 or 0.2 for DK180HV‐based (DoE 1 PU_opt_) and Desmodur Eco N7300‐based (DoE 2 PU_opt_) PUs, respectively (Table 3). DoE 1 PU_opt_ and DoE 2 PU_opt_ are compared to both the commercial PU and PU_opt_ (based on DK180HV isocyanate and Hg‐catalyst developed in our previous work).^[3]^ Particularly, transmittance values at 555 nm for both DoE 1 PU_opt_ (89.6 %) and DoE 2 PU_opt_ (89.3 %) slightly outperform the commercial polyurethane (T%≥88.0 %). Of note, the UV‐Vis spectra of the latter revealed a slight shift of the absorption cut‐off towards lower wavelengths (Figure 3). This is likely ascribable to the higher amount of LCR540RT in the commercial formulation compared to our PU_opt_, in which Sovermol780 is also used as polyol: as shown in Figure S8, LCR540RT has an absorption cut‐off at 330 nm, much higher in energy compared to the one of Sovermol780 (at 380 nm).

Both DoE 1 PU_opt_ and DoE 2 PU_opt_ show comparable gel‐time values (78 and 80 minutes, respectively) to commercial polyurethane (70 minutes). In contrast, a discrepancy could be noted for T_g_ value: only DoE 2 PU_opt_ emulates the thermal behaviour of the commercial formulation (T_g_=35.3 °C vs 35.0 °C, respectively), whereas DoE 1 PU_opt_ shows a higher T_g_ (59 °C) comparable with PU_opt_ (55 °C). These expected trends can be rationalized by considering the hydroxyl number and %NCO of the different polyols and isocyanates. As is well known, in a crosslinked polyurethane, the T_g_ tends to increase with higher hydroxyl numbers and %NCO.^[64]^ In this context, the commercial polyurethane demonstrates a T_g_ of 35 °C, formulated from a polyol (LCR540RT) with a hydroxyl number of 320 mg KOH/g and an isocyanate (DK180HV) with an %NCO of 27 %. The polyol blend (10 % BHET, 70 % Sovermol780, 20 % LCR540RT) used in the optimized formulations (PU_opt_, DoE 1 PU_opt_, DoE 2 PU_opt_) shows a higher hydroxyl number compared to the commercial reference, equal to 466 mg KOH/g. Since PU_opt_ and DoE 1 PU_opt_ are formulated with DK180HV, an increase in T_g_ value compared to the commercial PU was expected. On the other hand, DoE 2 PU_opt_, formulated with Desmodur N 7300, which has a lower %NCO content (21.5 %), shows a reduction in T_g_ compared to the other optimized formulations, aligning the value with the commercial reference. Hence, it is worth noting that DoE 2 PU_opt_ not only appears the most suitable formulation to replace the commercial reference, but also shows an outstanding percentage of renewable and circular content of 65 % w/w.

As summarised in Table 3, both commercial PU and PU_opt_ resulted in bubbles‐free films, which have been related to using an Hg‐based catalyst. Conversely, despite the optimization of both C% and B/Z factors, PUs with Bi−Zn catalysts still exhibited a not sufficient optical purity, due to presence of few amount of bubbles. In this perspective, one strategy to achieve the Hg‐based PUs features could be the use of a lower catalyst concentration compared to the boundary set in this work. However, as evidenced by the preliminary tests and the Sweet Spot plots, a lower C% would lead to unsuitable longer gel times and not completely polymerization of PUs, jeopardizing the obtainment of a ready‐to‐market formulation. An alternative approach to obtain optical pure polyurethane films is the addition of a small amount of a specific long‐chain fatty acid (LCA) into the polyol mixture as a reactivity modulator. According to U.S. patent 10246545B2, carboxylic acids tend to increase the pot‐life of the polyurethane resin^[65]^ and the induction period, while limiting the heat release during the polymerization. At the same time, the selectivity towards the desired urethane reaction is preserved while minimizing the occurrence of side reactions, such as the formation of carbon dioxide into the PUs film.

One should note that, within the DoE investigations, we resolved not to add LCA as a factor to minimize the complexity of factor interactions and to reduce the number of experiments. As a matter of fact, the overall quality of both DoE 1 PU_opt_ and DoE 2 PU_opt_ demonstrates the potentiality to produce PU films with a remarkably high percentage of bio‐based precursors (up to 65 %) and no toxic catalyst, that closely match the characteristics of the commercial reference.

Both optimized formulations, (DoE 1 and 2 PU opt) were produced incorporating 1 % w/w of LCA (namely, DoE 1 PU_opt_ LCA and DoE 2 PU_opt_ LCA) into the polyol mixture. Due to the lower amount of catalysts and the higher percentage of green components within the formulation, DoE 2 PU_opt_ LCA was selected for an in‐depth characterization, to confirm that the addition of LCA, which clearly improves the optical quality of the film, has any negative impact on the characteristic of the final manufact. Here, as can be seen in Table 3 and Figure S9, the resulting PU film retains the desired properties of the LCA‐free formulations, while exhibiting an extremely high optical purity.

**Figure 4:**
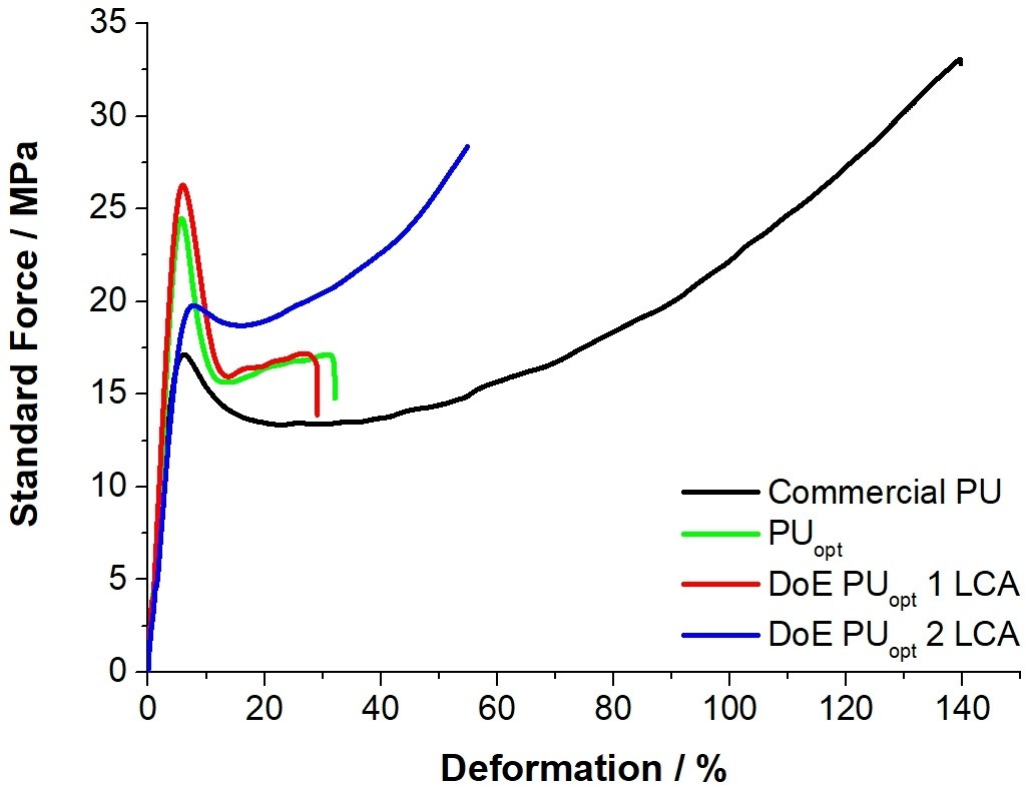
Mechanical properties of optimized Pus.

To further investigate and compare the final characteristics of the optimized PUs, mechanical and swelling analysis has been conducted. As shown in Figure 4 and reported in Table 4, the mechanical analyses reveal a similar stress‐deformation profile between PU_opt_ and DoE 1 PU_opt_ LCA. This indicates that replacing the mercury‐based catalyst with a more eco‐friendly alternative (i. e. the bismuth and zinc mixture) does not result in relevant changes in the mechanical behavior. Indeed, both materials exhibit higher initial resistance to deformation compared to the other two optimized PUs, with a yield point at higher stress values, followed by a notable drop, which suggests a rapid transition into plastic deformation.[^66^, ^67^] This behavior can be attributed to the higher rigidity and glass transition temperatures of these two specimens compared to their counterparts, i. e. both the DoE 2 PU_opt_ LCA and the commercial reference. The latter two, characterized by lower T_g_ values, display lower resistance to deformation. More interestingly, they exhibit strain‐hardening behavior after an initial phase of plastic yielding (blue and black profiles in Figure 4), where the material progressively strengthens under tension, likely due to the alignment of polymer chains.[^68^, ^69^] Consequently, the final stress surpasses the initial peak, demonstrating greater plastic deformation capacity without premature failure, as expected by the higher flexibility (lower T_g_). The abovementioned results proves DoE 2 PU_opt_ LCA to be the formulation which most closely emulates the commercial reference. One should note here that the slightly lower elongation at break, compared to the commercial reference, does not compromise its applicability in C.A.S.E. field.

**Table 4:** Mechanical and swelling properties of commercial and more eco‐friendly PU formulations.

Sample	Modulus [MPa]	F_max_ [N/mm^2^]	E Break [%]	Load B [N/mm^2^]	Swelling [%]	Gel [%]	Sol [%]
Commercial PU^[a]^	593.8±101.4	32.7±2.1	137.7±3.5	32.7±2.1	120.0±1.7	99.3±0.5	0.7±0.5
PU_opt_^[a]^	557.9±129.1	24.5±1.3	35.9±8	15.2±1.2	145.9±7	96.4±0.2	3.6±0.2
DoE 1 PU_opt_ LCA	689.8±155.7	26.8±0.6	32.8±4.2	14.4±1.6	161.8±3.3	95.6±0.4	4.4±0.4
DoE 2 PU_opt_ LCA	479.9±115.6	28.7±0.6	53.2±1.6	28.7±0.6	26.9±3.2	99.2±0.4	0.8±0.4

The analyses of the swelling and sol/gel fraction tests (Table 4) in dimethylformamide (DMF) confirm the similarities between PU_opt_ and DoE 1 PU_opt_ LCA, further demonstrating that replacing Hg‐based catalyst with a properly C% and ratio of Bi−Zn catalyst in the formulation does not significantly impact on the final properties of the PU film. Additionally, data in Table 4 reveal that the PU_opt_ and DoE 1 PU_opt_ LCA samples exhibit significantly higher swelling values (145.9 % and 161.8 %, respectively) compared to DoE 2 PU_opt_ LCA and reference samples. This could be rationalized considering the incomplete crosslinking of these materials as proved by the lower %Gel values (96.4 % and 95.6 %, respectively), highlighting the presence of a greater amount of soluble components in these matrix, which facilitates solvent permeation and swelling. Furthermore, it is important to highlight the outstanding swelling resistance demonstrated by DoE 2 PU_opt_ LCA (26.9 %), likely associated with the replacement of the fossil‐based isocyanate with the bio‐based alternative. Desmodur eco N7300, with a functionality of 3.7, may provide a higher number of crosslinking points per isocyanate molecule. This results in a tighter network, making the structure less permeable to solvents.

Such evidence seems to be in strict contrast with the relatively T_g_ and good flexibility. Typically, the higher crosslink density the higher T_g_ and the more rigid the film.[^70^, ^71^] Here, several hypotheses can be proposed considering the long hydrocarbon chains characteristic of the bio‐based isocyanate: indeed, the chemical structure of the network, where longer, more flexible chain segments exist between crosslinking nodes could account for the lower T_g._ ^[72]^ Moreover, the molecular structure of DoE 2 PU_opt_ LCA, with urethane groups distributed over a larger volume compared to the other polyurethanes (Table 5) promotes the establishment of less weak (such as Van der Waals) interactions between polymer chains, leading to a lower T_g_.^[73]^

**Table 5:** Polyurethane compositions and polyols:isocyanate ratios.

Sample	Catalyst	Polyol	Isocyanate	Pol:Isoc Ratio^[b]^
Commercial PU^[a]^	Hg	LCR540RT	DK180HV	100 : 100
PU_opt_^3^	Hg	10 % BHET, 20 % LCR540RT, 70 % Sovermol780^[b]^	DK180HV	100 : 129
PUs DoE 1	Bi+Zn		DK180HV	100 : 129
PUs DoE 2	Bi+Zn		Desmodur Eco N7300	100 : 162

^[a]^ Commercial PU reference, 100 % fossil‐based; ^[b]^ Calculated by weight.

In conclusion, based on the results presented in Tables 3 and 4, it can be claim that DoE 2 PU_opt_ LCA is not only the polyurethane with the highest percentage of bio‐based components, but also the formulation that most closely approximates the properties of the commercial reference. Furthermore, was also proved that the implementation of a minimal amount of LCA allows to produce bubble‐free, ready‐to‐market polyurethane film that faithfully replicates all critical features of the commercial reference,. Importantly, our approach achieves these benefits without any alterations to the production process of the entire system. Thus, our study demonstrates a significant advantage in seamlessly integrating these advancements into existing industrial manufacturing processes.

## Conclusions

Throughout this contribution, we exploited a multivariate chemometric approach to obtain highly sustainable and transparent thermosetting polyurethanes, either replacing (i) the toxic Hg‐based catalyst with a tailored Bi−Zn mixture and (ii) a commercial fossil‐based isocyanate with a bio‐based one. The two‐step optimization approach, in which the total amount of catalyst and the relative B/Z ratio were investigated, was designed to identify ready‐to‐market PU formulations. Aiming at this, the implementation of a CRM‐free catalyst and a bio‐based isocyanate would preserve the features of the commercial reference resin but with significantly improved eco‐friendly nature. Targeting the application as encapsulants for optoelectronic, some key parameters were monitored, among which the more critical ones are the film transmittance and the gel time, both closely related to the possible presence of bubbles, as well as the glass transition temperature. A critical analysis allowed us to clarify how the two factors (and their interactions) qualitatively and quantitatively impact the PU film properties. Additionally, the optimization of PU formulations allows to meet as closely as possible the industrial requirements. The PU film obtained exploiting the formulation parameters coming from the DoE 2 can perfectly emulate or even overcome different specifications of the commercial resin, showing a remarkably higher T% (approaching 90 % at 555 nm) and swelling behaviour (~27 % after 24 h in DMF) with comparable gel time (78 minutes), T_g_ (35.7 °C) and mechanical properties. It is important to recall that this achievement is coupled with a dramatic increase in the bio‐based and recycled contents, as high as 65 % w/w in the final PU formulation. The only issue that would impede a market entrance of the formulation, i. e. the insufficient optical purity due to the presence of both CO_2_ and air bubbles trapped within the film, was effectively overcome by adding a negligible amount (1 % w/w) of a specific long‐chain fatty acid. This study paves the way for further developing environmentally friendly and ready‐to‐market thermosetting polyurethanes for coating applications.

## Experimental Section

### Materials

LCR540RT (Demak Polymers) is a 100 % fossil‐based polyol (a mixture of polypropylene glycol triols and glycerin starter, with molecular weight of 450 and 750), trifunctionalized (f=3), with an hydroxyl number (n OH) of 320 mg KOH/g. Sovermol780 (BASF) is a branched polyether/polyester polyol with a 65 % bio‐based content, a functionality equal to 3 and with a n OH equal to 510 mg KOH/g. It was supplied by Sigma‐Aldrich. Bis(2‐hydroxyethyl) terephthalate (CAS 959‐26‐2) and dimethylformamide (DMF) (≥99 %, CAS 68‐12‐2) were supplied by Sigma‐Aldrich. DK180HV (Demak Polymers) is a prepolymerized polyisocyanate, composed of a mixture of isophorondisocyanate (IPDI) and IPDI prepolymer/linear polyester, with functionality equal to 2.2. Desmodur Eco N7300 (Covestro) is an aliphatic polyisocyanate (PDI‐trimer) with 71 % bio‐based content and functionality equal to 3.7. The organomercury‐based catalyst and the long‐chain fatty acid (LCA) were supplied by Demak Polymers. Organobismuth (Coscat® 83) and organozinc (Coscat® Z‐22R) catalysts were supplied by Vertellus.

### Polyurethane Resin Formulation and Film Preparation

The formulation of polyurethane resins involves the same steps followed in our previous work.^[3]^ Briefly, the polyol mixture composed by 70 % Sovermol780, 20 % LCR540RT, and 10 % BHET has been formulated by adding different amounts of bismuth‐ and zinc‐based catalysts, as expressly indicated by the DoE plan.

The polyol blend is stirred and heated at 70 °C for 30 minutes, then it is degassed under vacuum for 10 min, and finally mixed with the fossil‐based (DK180HV) or bio‐derived (Desmodur N7300) isocyanate using 1 : 1 stoichiometric ratio (hydroxyl:isocyanate groups) for 2 min at 70 °C. The preparation of polyurethane films involves a simple casting method, in which 5 g of PU resin is poured into a polypropylene mold and allowed to cool from 70 °C to room temperature. The cast resin is left to polymerize in the air for 24 hours, resulting in the formation of PU film with a ~0.8 mm thickness. Finally, the PU film is peeled off from the mold for characterization.

### Polyurethane Characterizations

The film transparency was evaluated by UV‐Vis spectrophotometry with an Agilent Cary 300 Bio spectrophotometer. The measurements were performed from 800 nm to 200 nm wavelengths in solid‐state mode using air as reference and the optical transmittance was evaluated at 555 nm.

Differential scanning calorimetry (DSC) analyses (Exo Up) were performed to evaluate the glass transition temperature (T_g_, analysed from the 2^nd^ heating curve), with a TA Instruments Q200 equipped with the RCS90 cooling system. Three measurement cycles (heating, cooling, and heating) were carried out in nitrogen with an isotherm of 5 minutes between each step. The heating ramps were set at 20 °C/min, up to 180 °C. The cooling ramp was set at 10 °C/min, up to −80 °C. The measurements were carried out in aluminium pans.

The gel time was measured with a Gel Instrument AG GELNORM geltimer at room temperature to evaluate the pot‐life of PU resin. Time zero is right after mixing polyols and isocyanate, and the time the formulations cease to flow was taken as the gel time value.^[61]^

Attenuated total reflectance infrared spectroscopy (FTIR‐ATR) was adopted to monitor the kinetic and the completeness of PU polymerization process. The measurements were realized in solid‐state mode with a Bruker Invenio IR, coupled with a Bruker Platinum ATR diamond, in a wavenumber range from 4000 cm^−1^ to 500 cm^−1^, with a scanning velocity of 20 kHz. PU curing was monitored by evaluating the ratio between the intensity of the isocyanate group (2270 cm^−1^) characteristic peak after 1 day and the intensity at t=0, as depicted in Figure S10.^[74]^ All the spectra were normalized for the intensity of the methylene absorption bands (at 2700–3000 cm^−1^), which remain unaltered during the entire curing process.^[75]^

The optical purity of the PUs was evaluated by monitoring the amount of bubbles trapped inside the cured film as a qualitative indicator. The latter is evaluated using a progressive and qualitative scale (Figure S3) due to the absence of a quantitative method for assessing their exact quantity: 0=no bubbles (ideal optical purity, considered the goal of the study); 1=low amount of bubbles; 2=moderate amount of bubbles; 3=high amount of bubbles; 4=very high amount of bubbles (almost 100 % of the entire surface covered with bubbles).

Viscosity characterizations were performed with a Brookfield DV−E viscometer at room temperature in a cylindrical vessel of 35 ml. The spindle number used for the measurement was S63 (largest spindle possible for the vessel), and the angular velocities (measured in Rate Per Seconds, RPM) were set as high as possible for each precursor and formulation.

Uniaxial tensile measurements were conducted on a Zwick Roell dinamometer, testcontrol II, equipped with a 5 kN load cell at room temperature. The specimens were cut into dog‐bone shapes with dimensions according to ASTM D638 type V. The tests were conducted using a 5 mm/min displacement after applying a preload force of 1 N. For each sample, five specimens were tested and the values were averaged.

Polymer swelling was studied by monitoring the weight variation of PU films when immersed in DMF. PU films were placed in a closed vial with solvent (30 mL) at room temperature. The measurements were carried out after 24 h by removing the sample from solvent and fast drying the excess of solvent with paper towel. The percentage of swelling (S%) was calculated using equation 9:

(9) $$S\%={\left(\frac{W-W_{0}}{W_{0}}\right)}^{*}100$$

where W_0_ and W are the sample weights before and after swelling, respectively. The samples were then dried in a vacuum oven for 24 h at 120 °C and weighed to assess the gel fraction (y) and the loss in weight (sol fraction) (Eq. 10 and 11:
(10)

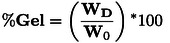

(11) %Sol=100-%Gel

Where W_D_ is the weight of the sample after drying.

#### Design of Experiments

The investigation of the newly proposed polyurethane formulations was guided by a multivariate chemometric approach based on the Design of Experiments, using MODDE Pro software (version 12.1), from Sartorius.[^80^, ^82^, ^83^] Response surface modelling design has been adopted and two similar DoEs have been performed to study the substitution of Hg‐catalyst in the polyurethane resins formulated with the fossil‐based isocyanate (DoE 1) and the bio‐derived isocyanate (DoE 2). The effect of different bismuth/zinc catalyst ratios (B/Z) and their total amount (sum of bismuth and zinc, C%) in the PU formulations were selected as the experimental variables (factors) of DoEs, as reported in Table 6.

**Table 6:** Factors and their selected operative ranges for DoE 1 and DoE 2.

Factors	Abbreviation	Ranges	Units
Bi/Zn catalyst ratio	B/Z	0.1–0.5	w/w
Catalysts %	C%	0.1–0.8	%

The effects of these factors on the final properties of PU films have been evaluated by measuring four different responses, selected to monitor (and possibly emulate) key features characterizing the commercial reference polyurethane (containing fossil‐based polyol and isocyanate with Hg‐catalyst): (i) gel time, (ii) transmittance at 555 nm (T%), (iii) amount of bubbles, and (iv) glass transition temperature (T_g_). Furthermore, FTIR‐ATR measurements have been conducted one day after formulation solely to confirm the completion of polymerization and they will not be discussed further. Here it should be noted that the absence of the characteristic isocyanate absorption band at 2270 cm^−1^ established the completion of the curing process (Figure S10).

As reference, commercial polyurethane is characterized by high transmittance, optical purity, an almost complete polymerization after one day and a long enough induction period with suitable gel time to ensure the processability of the resin.

After factors and responses selection, an optimization method based on a Full Factorial design has been selected for both DoEs to investigate the interactions and the quadratic terms of factors.[^76^, ^77^] According to the selected model, a certain experimental response (*y*) is described by Eq. 12:

(12) $$y=b_{0}+b_{1}x_{1}+b_{2}x_{2}+b_{12}x_{1}x_{2}+b_{11}x_{1}^{2}+b_{22}x_{2}^{2}\;$$

where $x_{1}$ and $x_{2}\;$ represent the experimental variables, while $b_{0}$ , $b_{1}$ , … are the coefficients that quantify the influence of each factor and their interactions on the response. A multiple linear regression model was computed for each response.[^78^, ^79^]

The goodness‐of‐fit of the computed model, which indicates how well it fits a set of experimental observations, and the model predictive power, which describes the ability to generalize to new, unseen data, have been evaluated by R^2^ and Q^2^ statistical parameters, respectively. The analysis of variance (ANOVA) test, the Observed vs. Predicted plot, and Residuals vs. Run Order plot were also considered for model diagnostics. The ANOVA compares the regression model and residuals to assess if the model is statistically significant at 95 % confidence level. The Observed vs. Predicted plot shows the observed experimental values versus the predicted values of response. Plots with points close to a straight line indicate good models. The Residuals vs. Run Order plot shows the deleted studentized residuals versus the run order of experiments and helps to detect any dependency of the residuals on time. Further details on the mathematical calculation and statistics used by Modde software are available online, in the MODDE User Guide.^[80]^

The final validation of the models was assessed by comparing the experimental result with the response value predicted by the model in a randomly selected experimental point (not considered in the DoE analysis). If the experimental value falls within the confidence intervals computed by the software, the model is deemed valid. It should be noted that, if required, the response values have been transformed to achieve a “bell shaped” normal distribution, leading to enhanced model estimation and statistic.

## Supporting Information

In the supporting information file, (i) the spectroscopic characterization of selected precursors and resin, as well as (ii) their thermal characterization. Additionally (iii) some digital photographs of the PU obtained in the screening phase, (iv) the structure of the two di‐isocyanates and (v) the sweet‐spot plots are included.

## Conflict of Interests

There are no conflicts to declare.

## Supporting information

As a service to our authors and readers, this journal provides supporting information supplied by the authors. Such materials are peer reviewed and may be re‐organized for online delivery, but are not copy‐edited or typeset. Technical support issues arising from supporting information (other than missing files) should be addressed to the authors.

Supporting Information

## Data Availability

The data that support the findings of this study are available from the corresponding author upon reasonable request.
