# Supplementary material for: Eco‐Friendly and Ready‐To‐Market Polyurethanes: A Design of Experiment‐Guided Substitution of Toxic Catalyst and Fossil‐Based Isocyanate
Source: ChemSusChem. 2025 Jan 17;18(12):e202402451. doi: 10.1002/cssc.202402451 (PMC12175042; doi:10.1002/cssc.202402451)
Supplement: Supplementary file 1 — Supporting Information [file CSSC-18-e202402451-s001.pdf]

ChemSusChem

Supporting Information

# Eco-Friendly and Ready-To-Market Polyurethanes: A Design of Experiment-Guided Substitution of Toxic Catalyst and Fossil-Based Isocyanate

Gabriele Viada, Nicole Mariotti, Simone Galliano,* Alberto Menozzi, Claudia Barolo, and Matteo Bonomo*

# Supporting Information of

# Eco-friendly and ready-to-market polyurethanes: a Design of Experiment-guided substitution of toxic catalyst and fossil-based isocyanate

Gabriele Viada,[a] Nicole Mariotti,[a] Simone Galliano,*[a] Alberto Menozzi,[c] Claudia Barolo,[a,b] Matteo Bonomo*[a]

[a] Mr. G. Viada, Dr. N. Mariotti, Dr. S. Galliano, Prof. C. Barolo, Dr. M. Bonomo
Department of Chemistry, NIS Interdepartmental Centre and INSTM Reference Centre
University of Turin
Via G. Quarello 15A, 10135 Torino, Italy.

[b] Prof. C. Barolo
Institute of Science, Technology and Sustainability for Ceramics
National Research Council of Italy
Via Granarolo 64, 48018, Faenza, Italy.

[c] Dr. A. Menozzi
Demak Polymers
Corso Lombardia 44,10151 Torino, Italy

Email: Matteo.bonomo@unito.it, simone.galliano@unito.it

**Figure S1**. Observed vs. Predicted plots showing the observed experimental values versus the predicted values for each response of DoE 1. Values for T% and $T_g$ responses were transformed by negative logarithm as reported in the manuscript.

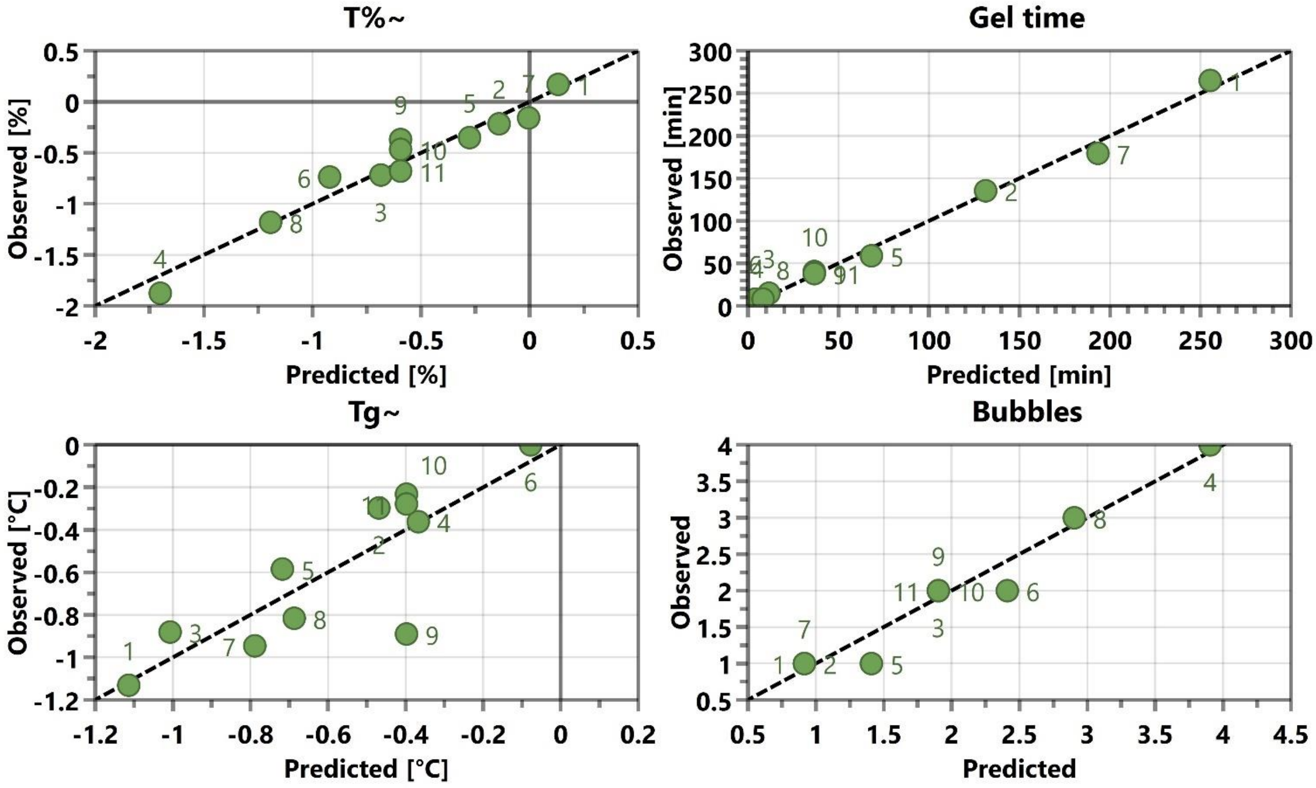

**Figure S2**. Deleted studentized residuals versus the run order of experiments for each response of DoE 1.

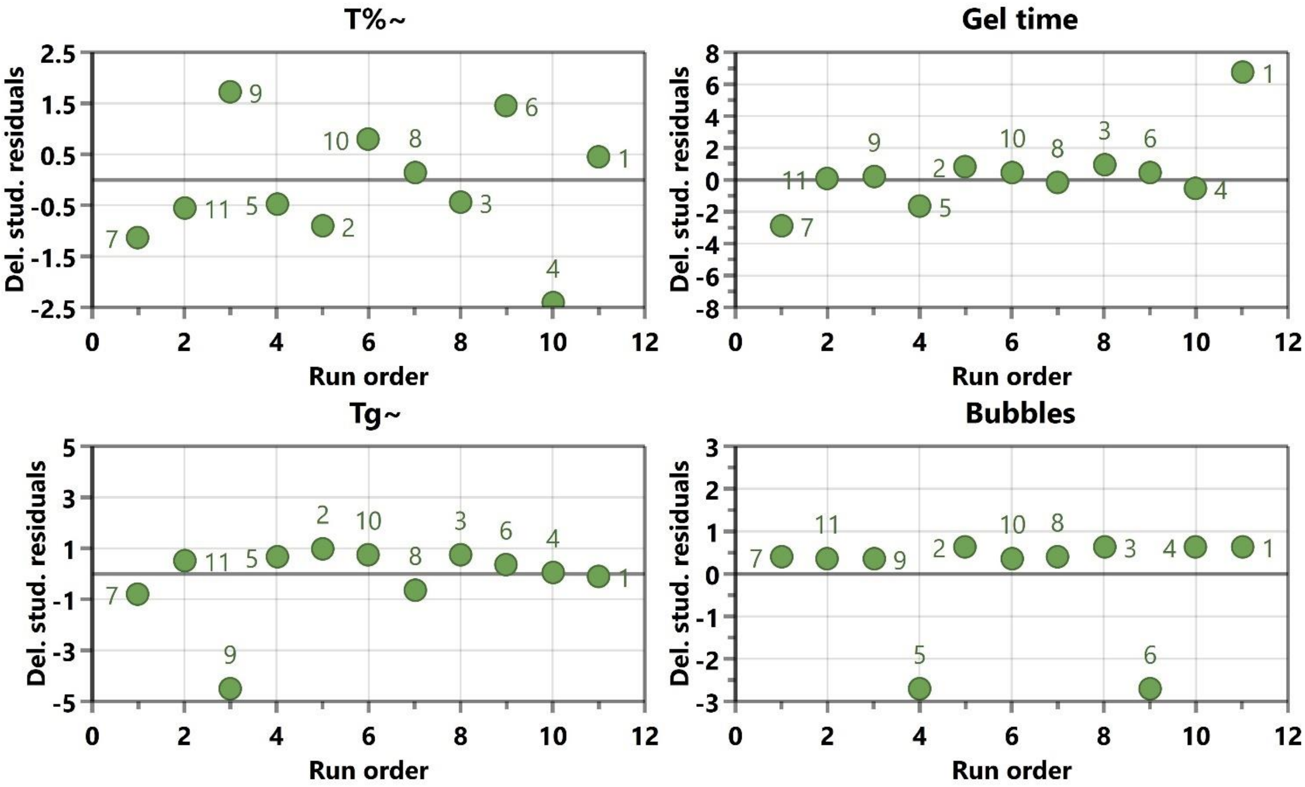

**Figure S3**: Qualitative scale for bubbles assessment. A = very high amount of bubbles (score = 4); b = high amount of bubbles (score = 3); c = moderate amount of bubbles (score = 2); d = low amount of bubbles (score = 1).

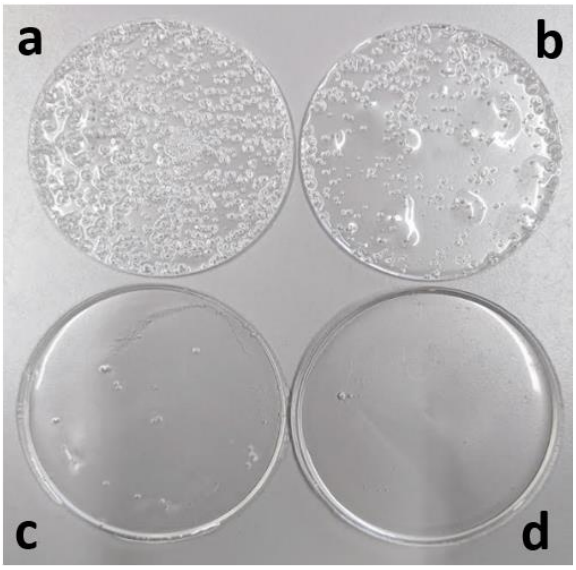

**Figure S4**. Observed vs. Predicted plots showing the observed experimental values versus the predicted values for each response of DoE 2.

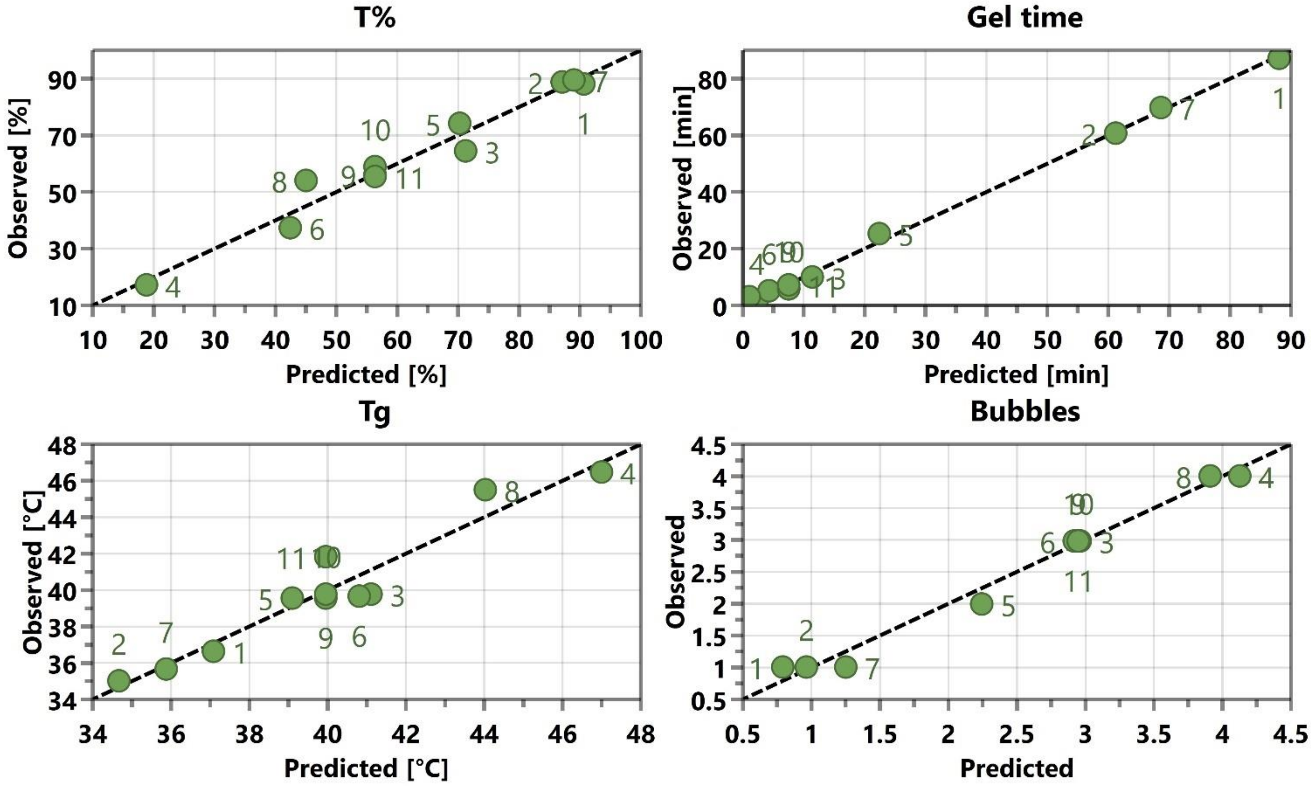

**Figure S5**. Deleted studentized residuals versus the run order of experiments for each response of DoE 2.

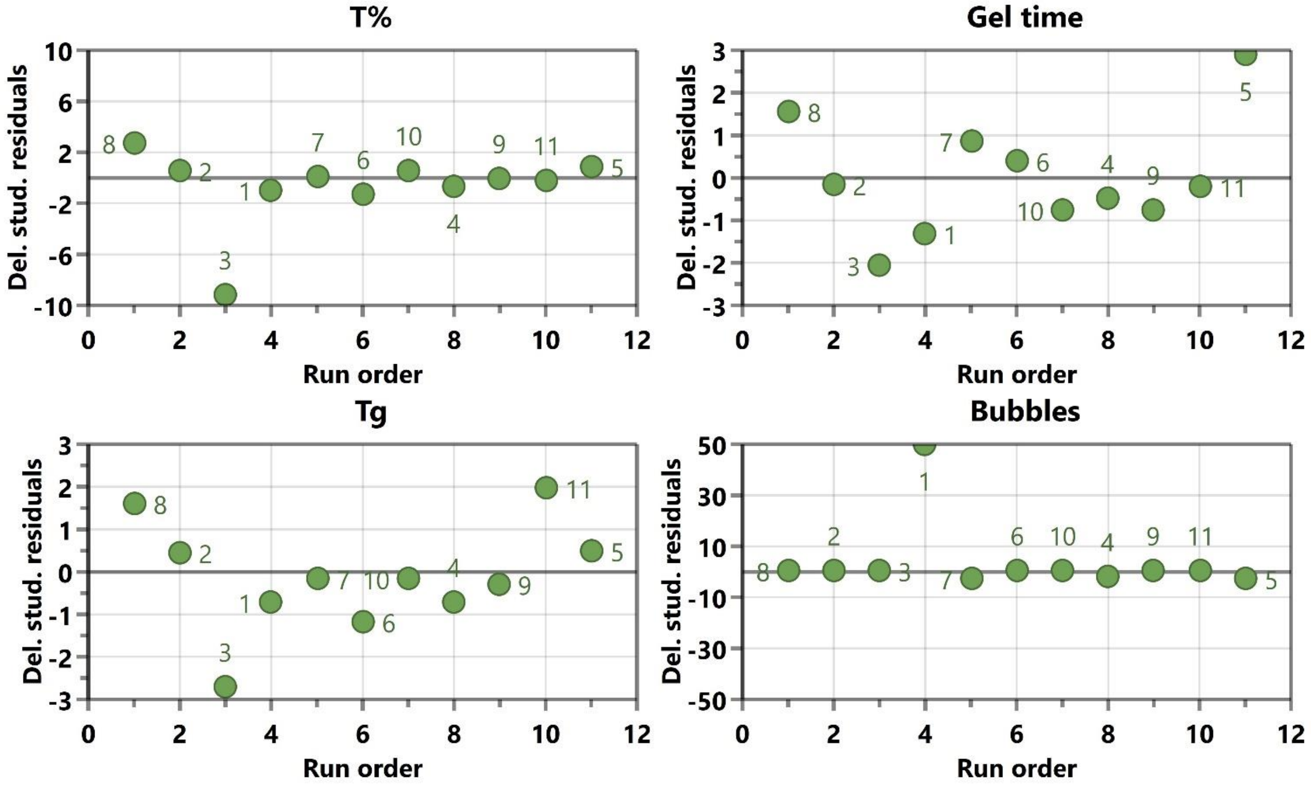

**Figure S6**. Chemical structures of isophorone diisocyanate (IPDI) (A) and Desmodur Eco N7300 isocyanate (B).

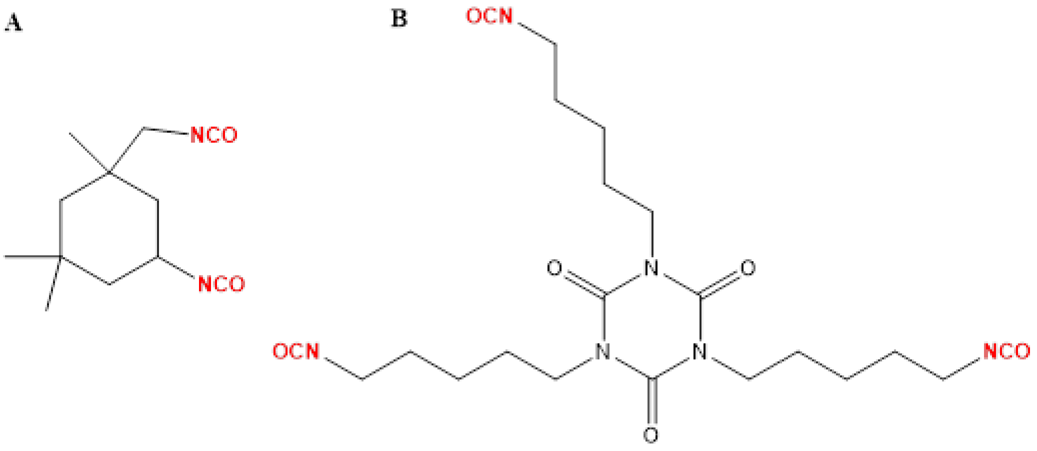

**Figure S7**: Sweet Spot plot for DoE 1 (up) and DoE 2 (down) investigation. Different colors highlight the number of met criteria: Blue for one criterion met, teal for two criteria met, and green for three criteria met.

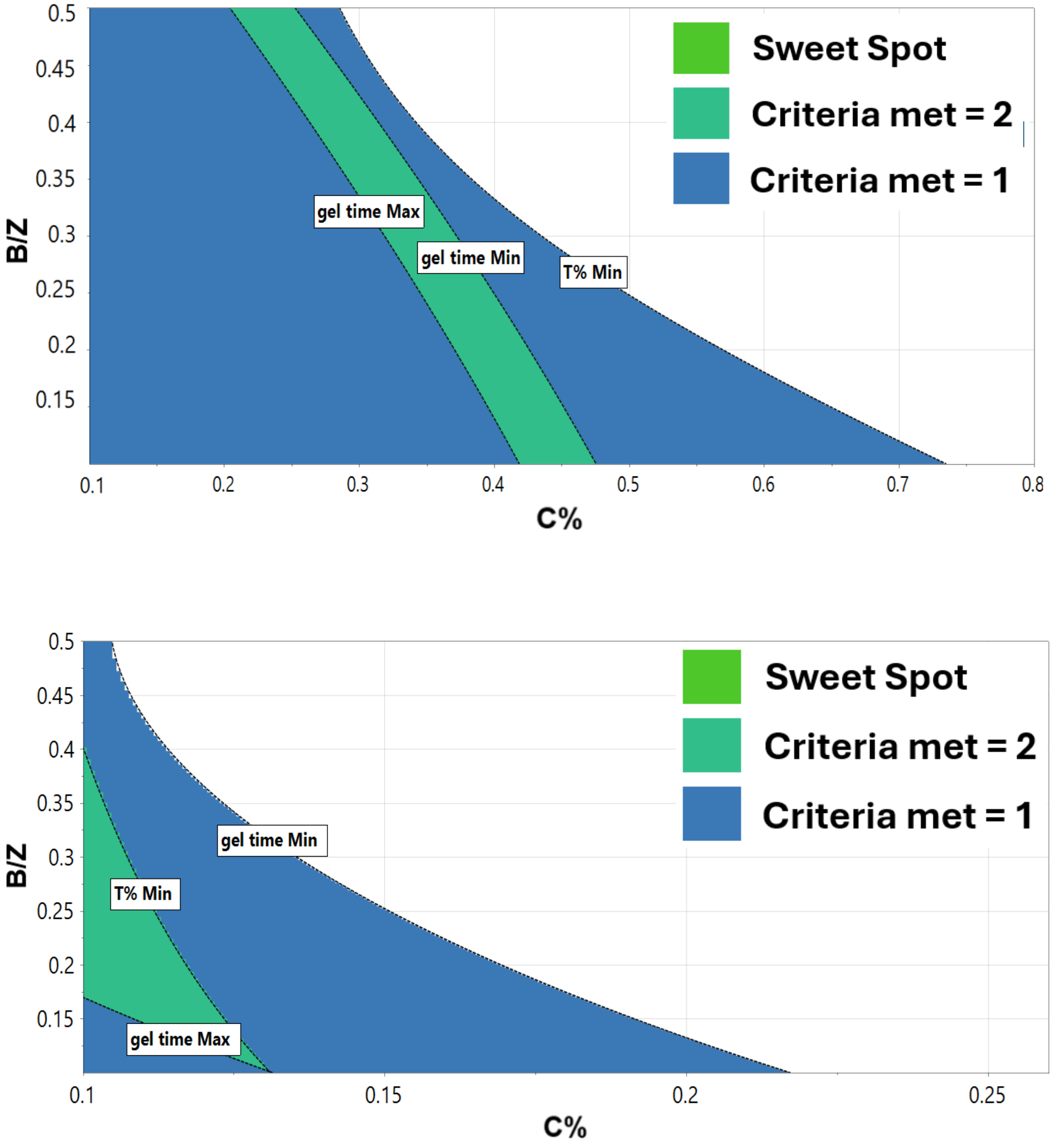

**Figure S8**. UV-Vis spectra of LCR540RT and Sovermol780 polyols without UV absorber.

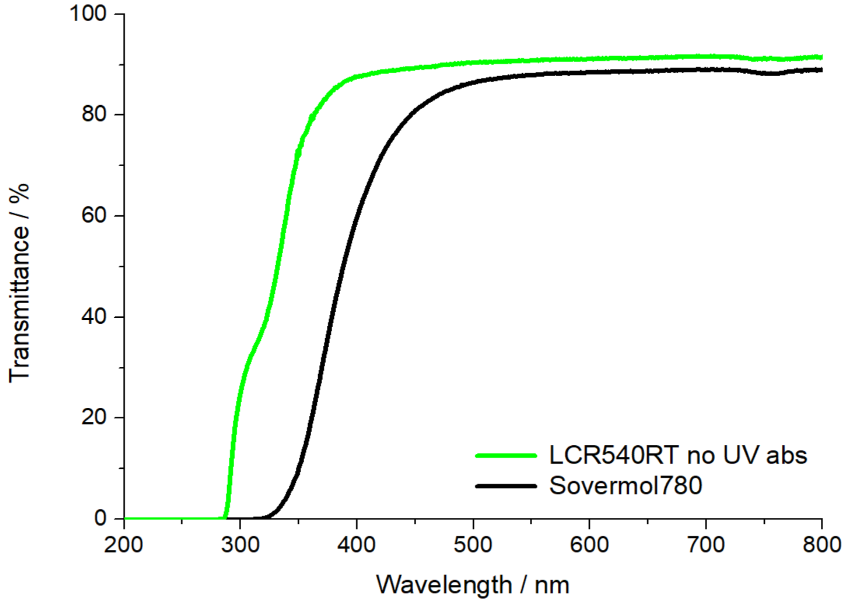

**Figure S9**. Differential scanning calorimetry (exo Up) of commercial PU and DoE 2 $PU_{opt}$ LCA.

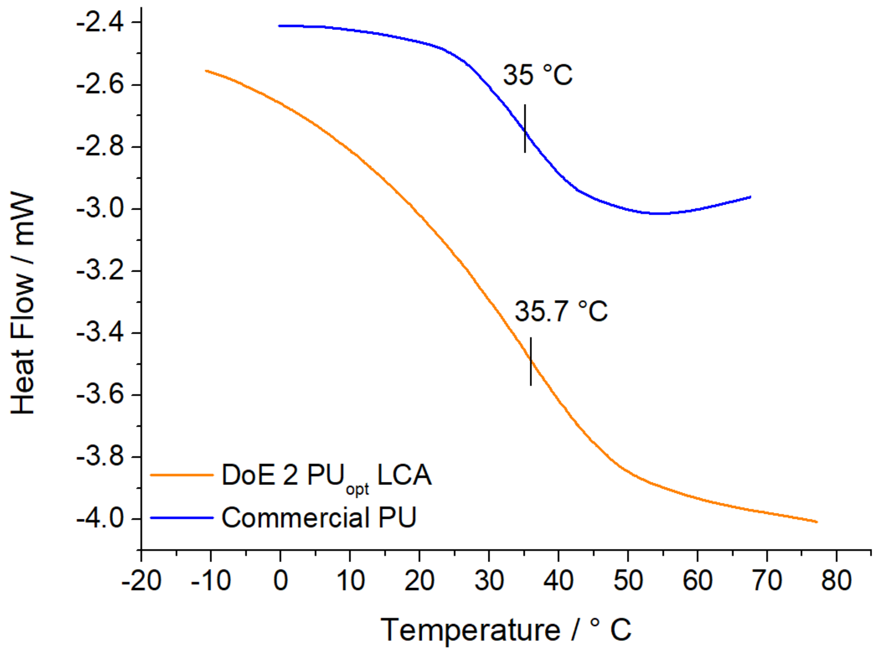

**Figure S10**. FTIR-ATR spectra normalization and isocyanate intensity monitoring.

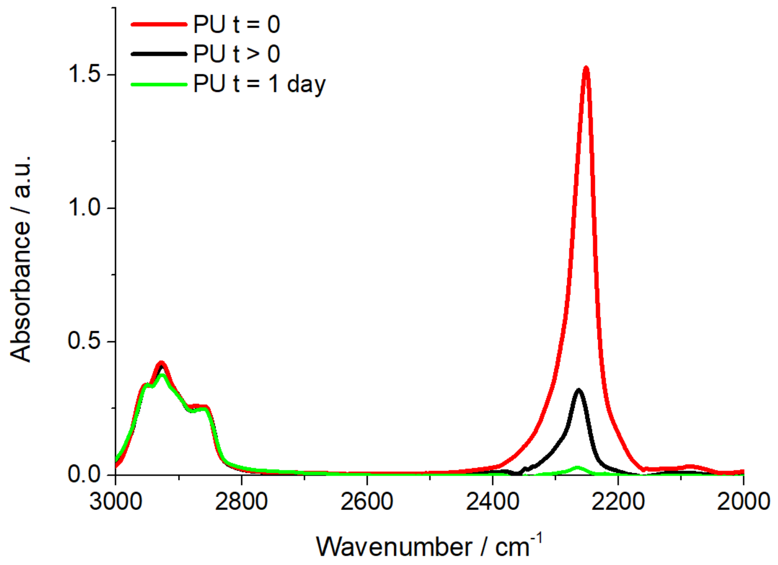

**Table S1**: Summarized ANOVA statistics from Modde software for DoE 1. (DF: Degree of Freedom; SS: Sum of Squares; MS: Mean Square; SD: Standard Deviation)

| T% | DF | SS | MS | F value | p-value | SD |
|---|---|---|---|---|---|---|
| Total | 11 | 6.98904 | 0.635367 | | | |
| Constant | 1 | 3.92921 | 3.92921 | | | |
| Total corrected | 10 | 3.05983 | 0.305983 | | | 0.553157 |
| Regression | 3 | 2.87935 | 0.959782 | 37.2248 | 1.1335e-04 | 0.979685 |
| Residual | 7 | 0.180484 | 0.0257834 | | | 0.160572 |

| Gel time | DF | SS | MS | F value | p-value | SD |
|---|---|---|---|---|---|---|
| Total | 11 | 129076 | 11734.2 | | | |
| Constant | 1 | 56163.3 | 56163.3 | | | |
| Total corrected | 10 | 72912.7 | 7291.27 | | | 85.3889 |
| Regression | 4 | 72484.3 | 18121.1 | 253.757 | 8.0814e-07 | 134.615 |
| Residual | 6 | 428.467 | 71.4112 | | | 8.45052 |

| $T_g$ | DF | SS | MS | F value | p-value | SD |
|---|---|---|---|---|---|---|
| Total | 11 | 5.09416 | 0.463105 | | | |
| Constant | 1 | 3.74203 | 3.74203 | | | |
| Total corrected | 10 | 1.35213 | 0.135213 | | | 0.367713 |
| Regression | 3 | 0.954315 | 0.318105 | 5.59742 | 2.8228e-02 | 0.564008 |
| Residual | 7 | 0.397814 | 0.0568306 | | | 0.238392 |

| Bubbles | DF | SS | MS | F value | p-value | SD |
|---|---|---|---|---|---|---|
| Total | 11 | 49 | 4.45455 | | | |
| Constant | 1 | 40.0909 | 40.0909 | | | |
| Total corrected | 10 | 8.90909 | 0.890909 | | | 0.94388 |
| Regression | 3 | 8.5 | 2.83333 | 48.4815 | 4.7432e-05 | 1.68325 |
| Residual | 7 | 0.409091 | 0.0584416 | | | 0.241747 |

**Table S2**. Model validation with experimental and predicted response values.

| | Validation points | | Experimental | | | | Predicted | | | |
|---|---|---|---|---|---|---|---|---|---|---|
| | **Cat% [%]** | **Bi/Zn** | **T% [%]** | **Gel time [min]** | **$T_g$ [°C]** | **Bubbles [a.u.]** | **T% [%]** | **Gel time [min]** | **$T_g$ [°C]** | **Bubbles [a.u.]** |
| DoE 1 | 0.77 | 0.40 | 65.8 | 5 | 63.2 | 3 | 68.7±9 | 3.0±12 | 62.0±2 | 3.3±0.4 |
| DoE 2 | 0.60 | 0.23 | 54.0 | 4.5 | 39.9 | 3 | 55.7±6 | 1.2±4 | 41.1±2 | 3.3±0.3 |

**Table S3**: List of optimized PU formulations computed from DoE 1 analysis.

| C% [%] | Bi/Zn | T% [%] | Gel time [min] | Bubbles [a.u.] |
|---|---|---|---|---|
| 0.42 | 0.10 | 89.3±0.9 | 79.5±12.5 | 1.3±0.3 |
| 0.21 | 0.50 | 88.7±1.7 | 77.5±14 | 1.4±0.4 |
| 0.31 | 0.32 | 88.7±0.8 | 79.1±8.6 | 1.5±0.2 |
| 0.41 | 0.11 | 89.3±0.9 | 81.5±12.2 | 1.4±0.3 |

**Table S4**: List of optimized PU formulation computed from DoE 2 analysis.

| C% [%] | Bi/Zn | T% [%] | Gel time [min] | Bubbles [a.u.] |
|---|---|---|---|---|
| 0.11 | 0.20 | 88.7±8.7 | 74.4±3.6 | 1.2±0.4 |
| 0.13 | 0.12 | 87.8±10.2 | 77.9±4 | 1±0.4 |
| 0.13 | 0.10 | 88.1±10.8 | 80.4±4.4 | 0.9±0.4 |
| 0.12 | 0.12 | 88.7±10.6 | 80.5±4.2 | 1±0.4 |

WILEY-VCH

**Table S5**: Summarized ANOVA statistics from Modde software for DoE 2 (DF: Degree of Freedom; SS: Sum of Squares; MS: Mean Square; SD: Standard Deviation)

| T% | DF | SS | MS | F value | p-value | SD |
|---|---|---|---|---|---|---|
| Total | 11 | 47664.6 | 4333.15 | | | |
| Constant | 1 | 42532.4 | 42532.4 | | | |
| Total corrected | 10 | 5132.26 | 513.226 | | | 22.6545 |
| Regression | 4 | 4947.61 | 1236.9 | 40.1922 | 1.8125e-04 | 35.1696 |
| Residual | 6 | 184.648 | 30.7747 | | | 5.54749 |

| Gel time | DF | SS | MS | F value | p-value | SD |
|---|---|---|---|---|---|---|
| Total | 11 | 17074 | 1552.18 | | | |
| Constant | 1 | 7229.45 | 7229.45 | | | |
| Total corrected | 10 | 9844.55 | 984.455 | | | 31.376 |
| Regression | 5 | 9823.69 | 1964.74 | 470.943 | 1.1202e-06 | 44.3254 |
| Residual | 5 | 20.8596 | 4.17192 | | | 2.04253 |

| $T_g$ | DF | SS | MS | F value | p-value | SD |
|---|---|---|---|---|---|---|
| Total | 11 | 17699.3 | 1609.03 | | | |
| Constant | 1 | 17568 | 17568 | | | |
| Total corrected | 10 | 131.265 | 13.1265 | | | 3.62306 |
| Regression | 3 | 121.771 | 40.5903 | 29.9256 | 2.3019e-04 | 6.37105 |
| Residual | 7 | 9.49462 | 1.35637 | | | 1.16463 |

| Bubbles | DF | SS | MS | F value | p-value | SD |
|---|---|---|---|---|---|---|
| Total | 11 | 84 | 7.63636 | | | |
| Constant | 1 | 71.2727 | 71.2727 | | | |
| Total corrected | 10 | 12.7273 | 1.27273 | | | 1.12815 |
| Regression | 5 | 12.5211 | 2.50423 | 60.7408 | 1.7823e-04 | 1.58247 |
| Residual | 5 | 0.20614 | 0.0412281 | | | 0.203047 |

WILEY-VCH
